# Metabolism of Dietary and Microbial Vitamin B Family in the Regulation of Host Immunity

**DOI:** 10.3389/fnut.2019.00048

**Published:** 2019-04-17

**Authors:** Ken Yoshii, Koji Hosomi, Kento Sawane, Jun Kunisawa

**Affiliations:** ^1^Laboratory of Vaccine Materials, Center for Vaccine and Adjuvant Research, and Laboratory of Gut Environmental System, National Institutes of Biomedical Innovation, Health and Nutrition, Osaka, Japan; ^2^Graduate School of Medicine, Osaka University, Osaka, Japan; ^3^Graduate School of Pharmaceutical Sciences, Osaka University, Osaka, Japan; ^4^Innovation Center, Nippon Flour Mills Co., Ltd., Atsugi, Japan; ^5^Graduate School of Dentistry, Osaka University, Osaka, Japan; ^6^Department of Microbiology and Immunology, Graduate School of Medicine, Kobe University, Hyogo, Japan; ^7^Division of Mucosal Vaccines, International Research and Development Center for Mucosal Vaccines, The Institute of Medical Science, The University of Tokyo, Tokyo, Japan

**Keywords:** absorption, gut microbiota, intestinal immunity, nutrition, vitamin

## Abstract

Vitamins are micronutrients that have physiological effects on various biological responses, including host immunity. Therefore, vitamin deficiency leads to increased risk of developing infectious, allergic, and inflammatory diseases. Since B vitamins are synthesized by plants, yeasts, and bacteria, but not by mammals, mammals must acquire B vitamins from dietary or microbial sources, such as the intestinal microbiota. Similarly, some intestinal bacteria are unable to synthesize B vitamins and must acquire them from the host diet or from other intestinal bacteria for their growth and survival. This suggests that the composition and function of the intestinal microbiota may affect host B vitamin usage and, by extension, host immunity. Here, we review the immunological functions of B vitamins and their metabolism by intestinal bacteria with respect to the control of host immunity.

## Introduction

The gut is continuously exposed both to toxic (e.g., pathogenic microorganisms) and beneficial (e.g., dietary components, commensal bacteria) compounds and microorganisms; therefore, the intestinal immune system must maintain a healthy balance between active and suppressive immune responses. This balance is controlled not only by host immune factors such as cytokines but also by a variety of environmental factors such as dietary components and the composition of the commensal bacteria. Furthermore, several lines of evidence have demonstrated that immune homeostasis in the intestine is related not only to intestinal health but also to the health of the whole body (1–3). Therefore, immune regulation by environmental factors is attracting attention as a means of maintaining immunological health and preventing many diseases.

Nutrients are essential for the development, maintenance, and function of the host immune system (4, 5). Vitamins are essential micronutrients that are synthesized by bacteria, yeasts, and plants, but not by mammals. Therefore, mammals must obtain vitamins from the diet or rely on their synthesis by commensal bacteria in the gastrointestinal tract. Some vitamins are water-soluble (e.g., vitamin B family and vitamin C), whereas others are fat-soluble (e.g., vitamins A, D, E, and K). Water-soluble vitamins are not stored by the body and any excess is excreted in the urine; therefore, it is important to consume a diet containing the necessary amounts of these vitamins. Vitamin deficiency associated with insufficient dietary intake occurs not only in developing countries but also in developed countries as a result of increased use of unbalanced diet (6).

In addition to the diet, the commensal bacteria are recognized as important players in the control of host health (7–9). From the point of view of vitamins, commensal bacteria are both providers and consumers of B vitamins and vitamin K. Although dietary B vitamins are generally absorbed through the small intestine, bacterial B vitamins are produced and absorbed mainly through the colon (10, 11), indicating that dietary and gut microbiota-derived B vitamins are possibly handled differently by the human body. B vitamins are important cofactors and coenzymes in several metabolic pathways, and it has been reported recently that B vitamins also play important roles in the maintenance of immune homeostasis (12, 13). Thus, both dietary components and the gut microbiota modulate host immune function via B vitamins. Here, we review the metabolism and function of dietary and gut microbiota-derived B vitamins in the control of host immunity.

## Vitamin B1

Vitamin B1 (thiamine) is a cofactor for several enzymes, including pyruvate dehydrogenase and α-ketoglutarate dehydrogenase, which are both involved in the tricarboxylic acid (TCA) cycle (14, 15). World Health Organization (WHO)/Food and Agriculture Organization (FAO) recommend a daily vitamin B1 intake of 1.1–1.2 mg for adult (16). Vitamin B1 deficiency causes lethargy and, if left untreated, can develop into beriberi, a disease that affects the peripheral nervous system and cardiovascular system. Accumulating evidence suggests that energy metabolism—particularly the balance between glycolysis and the TCA cycle—is associated with the functional control of immune cells, in what is now referred to as immunometabolism (17).

Immunometabolism is well studied in T cells and macrophages; quiescent or regulatory-type cells (e.g., naive T cells, Treg cells, and M2 macrophages) use the TCA cycle for energy generation, whereas activated or pro-inflammatory cells (e.g., Th1, Th2, Th17, and M1 macrophages) use glycolysis (18, 19).

**Figure 1:**
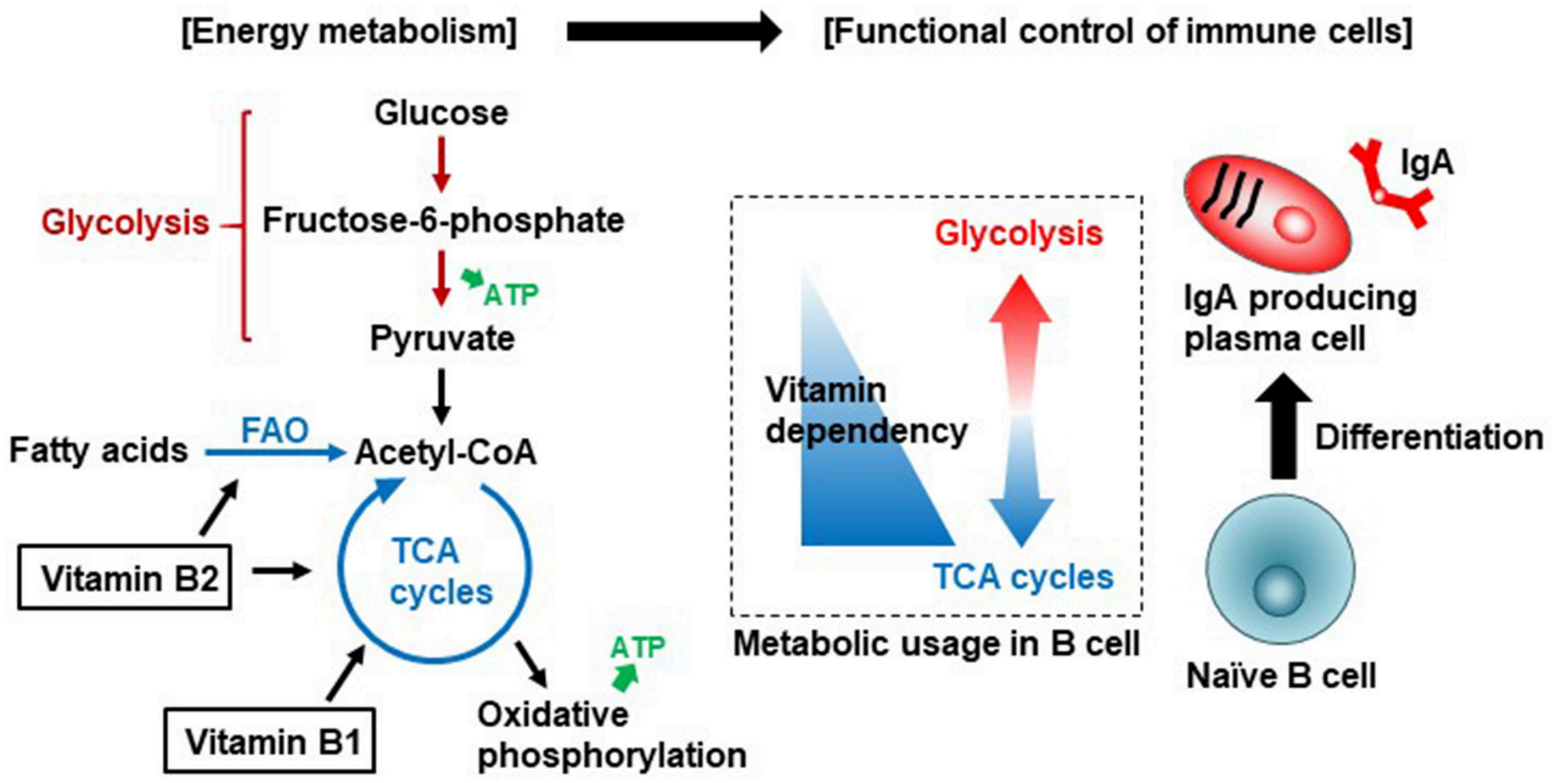
Vitamin B1 and B2-mediated immunometabolism in B cell differentiation in the intestine. Vitamin B1 acts as a cofactor for enzymes such as pyruvate dehydrogenase and α-ketoglutarate dehydrogenase that are involved in the TCA cycle. Vitamin B2 acts as a cofactor for enzymes such as succinate dehydrogenase in the TCA cycle and acyl-CoA dehydrogenase in fatty acid oxidation (FAO, also known as β-oxidation). Naïve B cells preferentially use the TCA cycle for efficient energy generation. Once B cells are activated to differentiate into IgA-producing plasma cells, they utilize glycolysis for the production of IgA antibody.

Previously, we examined B cell immunometabolism in the intestine. In the intestine, naïve immunoglobulin (Ig) M^+^ B cells differentiate into IgA^+^ B cells in Peyer's patches (PPs) by class switching, and then IgA^+^ B cells differentiate into IgA-producing plasma cells in the intestinal lamina propria (20). Naïve B cells in PPs preferentially use a vitamin B1-dependent TCA cycle for the generation of ATP. However, once the B cells differentiate into IgA-producing plasma cells, they switch to using glycolysis for the generation of ATP and shift to a catabolic pathway for the production of IgA antibody (Figure 1). Consistent with the importance of vitamin B1 in the maintenance of the TCA cycle, mice fed a vitamin B1-deficient diet show impaired maintenance of naïve B cells in PPs, with little effect on IgA-producing plasma cells. Since PPs are the primary sites of induction of antigen-specific IgA responses, PP regression induced by vitamin B1 deficiency leads to decreased IgA antibody responses to oral vaccines (21).

Vitamin B1 is found in high concentrations as thiamine pyrophosphate (TPP) in meat (particularly pork and chicken); eggs; cereal sprouts and rice bran; and beans. Dietary TPP is hydrolyzed by alkaline phosphatase and converted to free thiamine in the small intestine (22). Free thiamine is absorbed by the intestinal epithelium via thiamine transporters (e.g., THTR-1, THTR-2) and transported to the blood for distribution throughout the body (11). Free thiamine is converted back to TPP and is used for energy metabolism in the TCA cycle.

Various types of intestinal bacteria, mostly in the colon, also produce vitamin B1 as both free thiamine and TPP (11, 23). In the colon, free bacterial thiamine is absorbed mainly by thiamine transporters, transported to the blood, and distributed throughout the body; this mechanism is similar to how free dietary thiamine is taken up in the small intestine. However, unlike in the small intestine, TPP produced by the gut microbiota is not converted to free thiamine, because alkaline phosphatase is not secreted in the colon (24). Instead, TPP is absorbed directly by the colon via TPP transporters (e.g., TPPT-1) that are highly expressed on the apical membrane of the colon (25). The absorbed TPP enters the mitochondria via MTPP-1, a TPP transporter that is expressed in the mitochondrial inner membrane and is used as a cofactor for ATP generation (26). This suggests that bacterial TPP is important for energy generation in the colon. Thus, dietary and bacterial vitamin B1 appears to have different roles in the host.

The vitamin B1 structure consists of a thiazole moiety linked to a pyrimidine moiety. Bacteria obtain the thiazole moiety from glycine or tyrosine and 1-deoxy-d-xylulose-5-phosphate, and plants and yeasts synthesize it from glycine and 2-pentulose (27–30). In both bacteria and plants, the pyrimidine moiety is derived from 5-aminoimidazole ribonucleotide, an intermediate in the purine pathway (29). Metagenomic analyses of the human gut microbiota predict that *Bacteroides fragilis* and *Prevotella copri* (phylum Bacteroidetes); *Clostridium difficile*, some *Lactobacillus* spp., and *Ruminococcus lactaris* (Firmicutes); *Bifidobacterium* spp. (Actinobacteria); and *Fusobacterium varium* are vitamin B1 producers (Table 1) (10, 46), implying that many intestinal bacteria possess a complete vitamin B1 synthesis pathway, which includes pathways for the synthesis of thiazole and pyrimidine. Indeed, *Lactobacillus casei* produces thiamine during the production of fermented milk drinks (31), and *Bifidobacterium infantis* and *B. bifidum* produce thiamine in culture supernatant (32). However, *Faecalibacterium* spp. (Firmicutes) lack a vitamin B1 synthesis pathway even though they require vitamin B1 for their growth (10). Therefore, these bacteria must obtain their vitamin B1 from other bacteria or from the host diet via a thiamine transporter, suggesting that there is competition for vitamin B1 between the host and certain intestinal bacteria.

**Table 1:** Vitamin B family producing bacteria.

**Vitamins**	**Forms**	**Bacteria**	**References**
B1	Thiamin pyrophosphate (TPP)	*Bacteroides fragilis* *Prevotella copri* *Clostridium difficile* *Lactobacillus* casei *Lactobacillus curvatus* *Lactobacillus plantarum* *Ruminococcus lactaris* *Bifidobacterium infantis* *Bifidobacterium bifidum* *Fusobacterium varium*	(10, 31–33)
B2	Flavin adenine dinucleotide (FAD) Flavin mononucleotide (FMN)	*Bacteroides fragilis* *Prevotella copri* *Clostridium difficile* *Lactobacillus plantarum* *Lactobacillus fermentum* *Ruminococcus lactaris*	(10, 34–36)
B3	Nicotinic acid Nicotinamide	*Bacteroides fragilis* *Prevotella copri* *Ruminococcus lactaris* *Clostridium difficile* *Bifidobacterium infantis* *Helicobacter pylori* *Fusobacterium varium*	(10, 32)
B5	Free pantothenic acid	*Bacteroides fragilis* *Prevotella copri* *Ruminococcus lactaris* *Ruminococcus torques* *Salmonella enterica* *Helicobacter pylori*	(10)
B6	Pyridoxal phosphate (PLP)	*Bacteroides fragilis* *Prevotella copri* *Bifidobacterium longum* *Collinsella aerofaciens* *Helicobacter pylori*	(10, 32)
B7	Free biotin	*Bacteroides fragilis* *Lactobacillus helveticus* *Fusobacterium varium* *Campylobacter coli*	(10, 37)
B9	Tetrahydrofolate (THF)	*Bacteroides fragilis* *Prevotella copri* *Clostridium difficile* *Lactobacillus plantarum* *Lactobacillus delbrueckii* ssp. *bulgaricus* *Lactobacillus reuteri* *Streptococcus thermophilus Bifidobacterium pseudocatenulatum* *Bifidobacterium adolescentis* *Fusobacterium varium* *Salmonella enterica*	(10, 38–41)
B12	Adenosylcobalamin	*Bacteroides fragilis* *Prevotella copri* *Clostridium difficile* *Faecalibacterium prausnitzii* *Ruminococcus lactaris* *Propionibacterium freudenreichii* *Lactobacillus plantarum* *Lactobacillus coryniformis* *Lactobacillus s reuteri* *Bifidobacterium animalis* *Bifidobacterium infantis* *Bifidobacterium longum* *Fusobacterium varium*	(10, 32, 33, 42–45)

## Vitamin B2

Vitamin B2 (riboflavin) and its active forms (flavin adenine dinucleotide [FAD] and flavin mononucleotide [FMN]) are cofactors for enzymatic reactions in the TCA cycle and in fatty acid oxidization (also known as β-oxidization) (15). WHO/FAO recommends a daily vitamin B2 intake of 1.0–1.3 mg for adults (16). Vitamin B2 deficiency suppresses the activity of acyl-CoA dehydrogenases involved in the oxidation of fatty acids to generate acetyl-CoA, which is used by mitochondria to produce ATP via the TCA cycle. Fatty acid oxidization is involved in the activation, differentiation, and proliferation of immune cells through the generation of acetyl-CoA and its entry into TCA cycle (47). This suggests that vitamin B2 is associated with the control of differentiation and function of immune cells through regulation of fatty acid oxidization (Figure 1); however, the immunological roles of vitamin B2 in the control of host immunity remain to be investigated. In addition to energy generation, vitamin B2 is associated with reactive oxygen species (ROS) generation in immune cells through the priming of NADPH oxidase 2 (48); ROS are important effector and signaling molecules in inflammation and immunity.

Vitamin B2 is found at high levels in leafy green vegetables, liver, and eggs. Dietary vitamin B2 exists as FAD or FMN and is converted to free riboflavin by FAD pyrophosphatase and FMN phosphatase in the small intestine (49, 50). Free riboflavin is absorbed via riboflavin transporter expressed on the epithelium of the small intestine and is then released into the blood. In the blood, free riboflavin is converted back to FAD or FMN and distributed throughout the body (51–53).

Bacterial vitamin B2 is synthesized from guanosine triphosphate (GTP) and d-ribulose 5-phosphate (54). Bacterial vitamin B2 exists as free riboflavin, which is directly absorbed in the large intestine, converted to FAD or FMN, and distributed throughout the body as described above (23). A metagenome analysis of the human gut microbiota by Magnúsdóttir et al. (10) has predicted that *Bacteroides fragilis* and *Prevotella copri* (Bacteroidetes); *Clostridium difficile, Lactobacillus plantarum, L. fermentum*, and *Ruminococcus lactaris* (Firmicutes) express factors essential for vitamin B2 synthesis, suggesting that these bacteria are an important source of vitamin B2 in the large intestine (Table 1). In contrast, *Bifidobacterium* spp., and *Collinsella* spp. (Actinobacteria) lack a vitamin B2 pathway. Supplementation of fermented soymilk containing *Lactobacillus plantarum* with riboflavin deficient diet has been shown to promote vitamin B2 production and prevent vitamin B2 deficiency in mice (35). *L. fermentum* isolated from sourdough can synthesize riboflavin *in vitro* (36). Furthermore, recent evidence indicates that some species in Bacteroidetes phylum produce more riboflavin than do Actinobacteria and Firmicutes phyla (55). However, Actinobacteria and Firmicutes phyla still express riboflavin transporter and the enzymes necessary for FAD and FMN generation from free riboflavin (i.e., FAD synthases and flavin kinases) (10, 56), suggesting that all bacteria, including those that are unable to synthesize vitamin B2 themselves, require FAD and FMN for their growth and survival. Thus, as with vitamin B1, there is likely competition for riboflavin between the host and the commensal bacteria.

**Figure 2:**
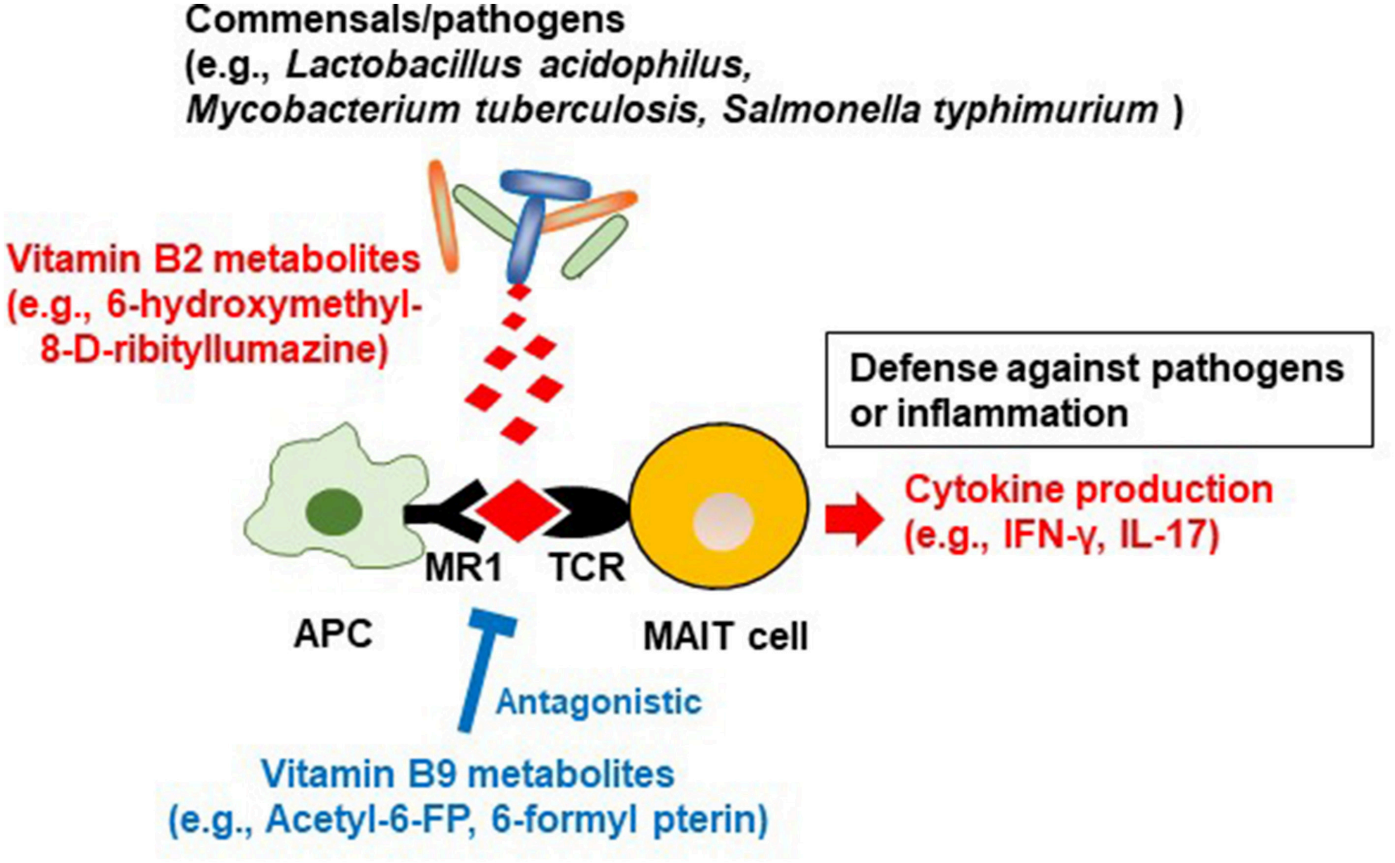
Regulation of MAIT cells by microbial metabolites derived from vitamin B2 and B9. Commensal bacteria/pathogens produce the vitamin B2 metabolite 6-hydroxymethyl-8-D-ribityllumazine. It binds to major histocompatibility complex (MHC) related protein (MR1) on antigen-presenting cells, which activate mucosal associated invariant T (MAIT) cells to promote the production of inflammatory cytokines such as IFN-γ and IL-17. These reactions contribute to defense against pathogens and conversely are associated with inflammation. In contrast, the vitamin B9 metabolite acetyl-6-formylpterin binds as an antagonist to MR1, thus inhibiting the activation of MAIT cells.

In addition to being able to produce vitamin B2, some bacteria (e.g., commensals such as *Lactobacillus acidophilus* and pathogens such as *Mycobacterium tuberculosis* and *Salmonella typhimurium*) produce the vitamin B2 intermediate (57–59), 6-hydroxymethyl-8-d-ribityllumazine (60, 61). 6-Hydroxymethyl-8-d-ribityllumazine binds to major histocompatibility complex class I-related gene protein (MR1) on antigen-presenting cells; this causes mucosal-associated invariant T (MAIT) cells, an abundant population of innate-like T cells, to produce cytokines such as interferon gamma and interleukin (IL) 17, which contribute to host defense against pathogens (Figure 2) (62). It is thought that stimulation by commensal bacteria contributes to the development and activation of MAIT cells for immunological surveillance against pathogens. MAIT cells also produce inflammatory cytokines and have tissue-homing properties, suggesting that these cells are also involved in the development of autoimmune and inflammatory diseases (63).

## Vitamin B3

Vitamin B3 (niacin) is generally known as nicotinic acid and nicotinamide. These compounds are precursors of nicotinamide adenine dinucleotide (NAD), a coenzyme in the cellular redox reaction with a central role in aerobic respiration. WHO/FAO recommends a daily vitamin B3 intake of 11–12 mg for adults (16).

**Figure 3:**
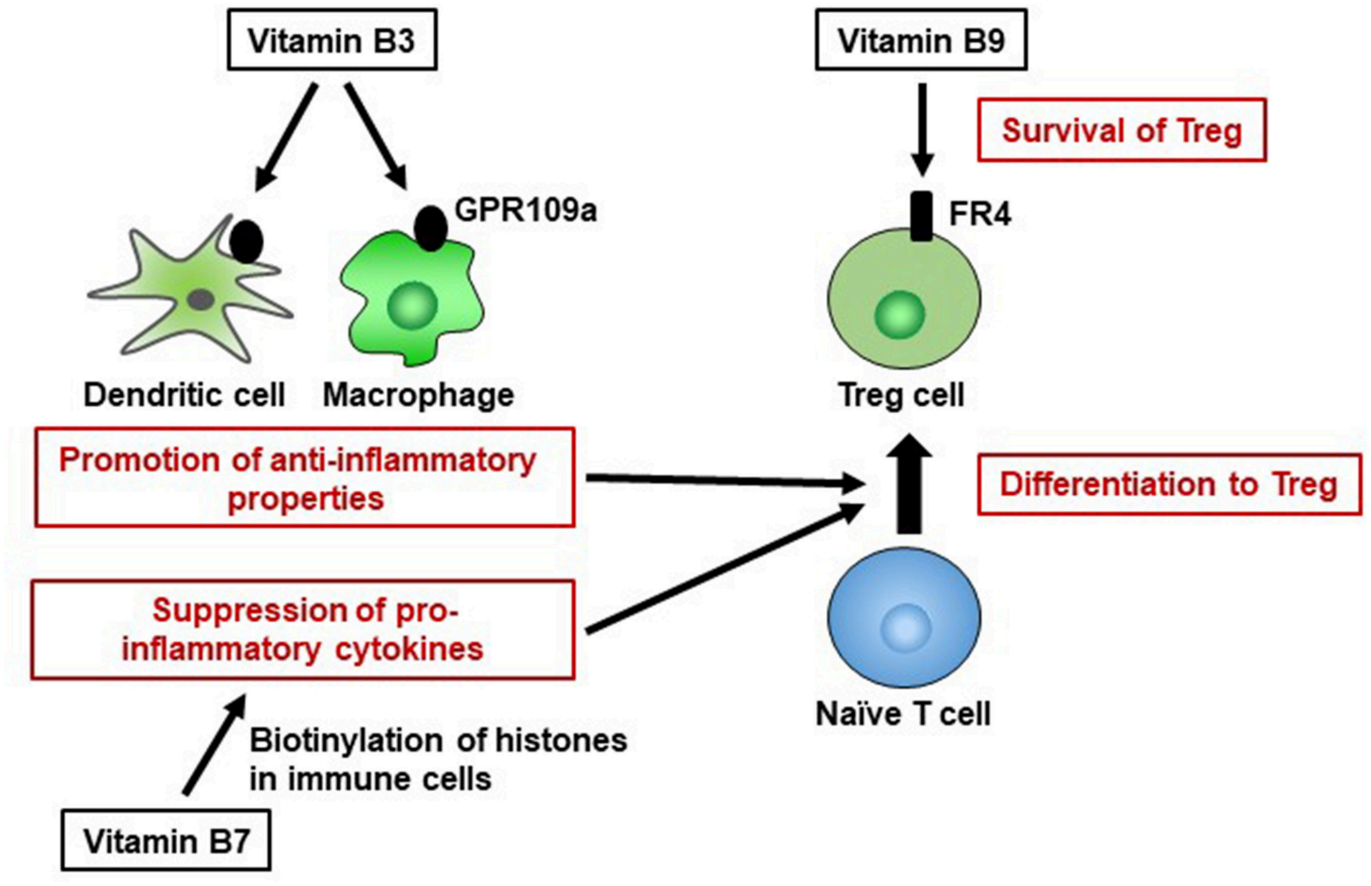
Pivotal roles of vitamins B3, B7, and B9 in maintenance of immunological homeostasis. Vitamin B3 binds to GPR109a in dendritic cells and macrophages, and GPR109a signaling leads to an increase in anti-inflammatory properties, resulting in differentiation into regulatory T cells (Treg). Vitamin B7 binds to histones and, by histone biotinylation, suppresses the secretion of pro-inflammatory cytokines. Once naïve T cells differentiate into Treg cells, they highly express folate receptor 4 (FR4). Consistent with this finding, vitamin B9 is required for the survival of Treg cells.

Vitamin B3 is also a ligand of GPR109a, a G-protein coupled receptor expressed on several types of cells, including immune cells (64). Vitamin B3–GPR109a signaling induces differentiation of regulatory T cells and suppresses colitis in a GPR109a-dependent manner (65). Vitamin B3 also inhibits the production of the pro-inflammatory cytokines IL-1, IL-6, and tumor necrosis factor alpha (TNF-α) by macrophages and monocytes (Figure 3) (66). Thus, vitamin B3 has anti-inflammatory properties by modulating host immune cells and playing an important role in the maintenance of immunological homeostasis. Indeed, in humans, vitamin B3 deficiency causes pellagra, which is a disease characterized by intestinal inflammation, diarrhea, dermatitis, and dementia (67).

Unlike the other B vitamins, vitamin B3 can be generated by mammals via an endogenous enzymatic pathway from tryptophan and is stored in the liver, although it is also obtained from the diet (68). Animal-based foods such as fish and meat contain vitamin B3 as nicotinamide, and plant-based foods such as beans contain vitamin B3 as nicotinic acid. Both nicotinamide and nicotinic acid are directly absorbed through the small intestine, where nicotinic acid is converted to nicotinamide.

Vitamin B3 is also synthesized from tryptophan by intestinal bacteria (69, 70). *Bacteroides fragilis* and *Prevotella copri* (Bacteroidetes); *Ruminococcus lactaris, Clostridium difficile* (Firmicutes); *Bifidobacterium infantis* (Actinobacteria); *Helicobacter pylori* (Proteobacteria); and *Fusobacterium varium* (Fusobacteria) possess a vitamin B3 biosynthesis pathway (Table 1) (10, 71). Thus, many intestinal bacteria from various genera can produce vitamin B3, suggesting that both dietary and commensal bacteria-derived vitamin B3 are important for host immunity.

## Vitamin B5

Vitamin B5 (pantothenic acid) is a precursor of coenzyme A (CoA), which is an essential cofactor for the TCA cycle and fatty acid oxidation (72). WHO/FAO recommends a daily vitamin B5 intake of 5.0 mg for adults (16). Like vitamins B1 and B2, vitamin B5 is involved in the control of host immunity via energy generation by immune cells. Vitamin B5 deficiency causes immune diseases such as dermatitis, as well as non-immune-related conditions such as fatigue and insomnia (73). In a randomized, double-blind, placebo-controlled study in adults, dietary supplementation with vitamin B5 was shown to improve facial acne (74), suggesting that epithelial barrier function improves via the promotion of keratinocyte proliferation and differentiation into fibroblasts (75). To maintain vitamin B5 levels during times of deficiency, CoA is converted back to vitamin B5 or cysteamine via pantetheine (76). However, cysteamine inhibits peroxisome proliferator-activated receptor gamma (PPARγ) signaling, causing inflammation (77). Indeed, colitis has been improved in pantetheine-producing-enzyme knockout mice (78). Thus, vitamin B5 deficiency causes inflammation through both dysfunction of the epithelial barrier and the production of pro-inflammatory molecules.

In terms of immune responses, vitamin B5 enhances protective activity against *Mycobacterium tuberculosis* infection by promoting innate immunity and adaptive immunity. In mice, vitamin B5 supplementation activates phagocytosis and cytokine production (including IL-6 and TNF-α) by macrophages and subsequently promotes Th1 and Th17 responses for the clearance of *M. tuberculosis* from the lungs (79). Thus, vitamin B5 contributes to host defense by activating immune responses, suggesting that this vitamin has an important role as a natural adjuvant.

Vitamin B5 is found in high concentrations as CoA or phosphopantetheine in liver, eggs, chicken, and fermented soybeans. CoA and phosphopantetheine are converted to free pantothenic acid by endogenous enzymes such as phosphatase and pantetheinase in the small intestine. Free pantothenic acid is absorbed via sodium-dependent multivitamin transporter (SMVT) expressed on the epithelium of the small intestine and is then released into the blood (80). Finally, free pantothenic acid is converted back to CoA and distributed throughout the body, particularly to the liver and kidney.

Bacterial vitamin B5 is synthesized from 2-dihydropantoate and β-alanine via *de novo* synthesis pathways (10). Bacterial vitamin B5 exists as free pantothenic acid, which is directly absorbed in the large intestine, converted to CoA, and distributed in the same way as dietary vitamin B5. According to a genomic analysis, *Bacteroides fragilis* and *Prevotella copri* (Bacteroidetes); some *Ruminococcus* spp. (*R. lactaris* and *R. torques*) (Firmicutes); *Salmonella enterica* and *Helicobacter pylori* (Proteobacteria) possess a vitamin B5 biosynthesis pathway, indicating that intestinal commensal bacteria can produce vitamin B5. In contrast, most *Fusobacterium* (Fusobacteria) and *Bifidobacterium* spp. (Actinobacteria) and some strains of *Clostridium difficile, Faecalibacterium* spp., and *Lactobacillus* spp. (Firmicutes) lack such a pathway (Table 1), although some of them do express pantothenic acid transporter to utilize vitamin B5 for energy generation (10), suggesting that these bacteria compete with the host for vitamin B5.

## Vitamin B6

Vitamin B6 exists in several forms, including as pyridoxine, pyridoxal, and pyridoxamine. These forms of vitamin B6 are precursors of the coenzymes pyridoxal phosphate (PLP) and pyridoxamine phosphate (PMP), which are involved in a variety of cellular metabolic processes, including amino acid, lipid, and carbohydrate metabolism (81). WHO/FAO recommends a daily vitamin B6 intake of 1.3–1.7 mg for adults (16). Vitamin B6 deficiency is associated with the development of inflammatory diseases such as allergy and rheumatoid arthritis, as well as with neuronal dysfunction (82–84). Vitamin B6 deficiency disrupts the Th1–Th2 balance toward an excessive Th2 response, resulting in allergy (85). Moreover, low plasma vitamin B6 levels, together with increased levels of pro-inflammatory cytokines such as TNF-α and IL-6, have been observed in patients with rheumatoid arthritis (86). However, the mechanism underlying the regulation of inflammation by vitamin B6 is currently unknown. Vitamin B6 contributes to intestinal immune regulation through the metabolism of the lipid mediator sphingosine 1-phosphate (S1P). S1P regulates lymphocyte trafficking into the intestines, especially in the large intestine. Lymphocyte trafficking is dependent on S1P gradient which is created by S1P production and its degradation. S1P degradation is mediated by S1P lyase that requires vitamin B6 as a cofactor. The administration of vitamin B6 antagonist impairs S1P lyase activity and creates an inappropriate S1P gradient, resulted in impairing lymphocyte migration from lymphoid tissues and reducing the numbers of lymphocytes in the intestines (87). The lymphocytes located between gut epithelial cells are known as intraepithelial cells (IELs) which are involved in the protection against pathogens (88). Therefore, vitamin B6 is important role for immunosurveillance in the intestines.

Vitamin B6 is abundant in fish, chicken, tofu, sweet potato, and avocado. Dietary vitamin B6 exists as PLP or PMP; it is converted to free vitamin B6 by endogenous enzymes such as pyridoxal phosphatase and is then absorbed by the small intestine. Although absorption of vitamin B6 through acidic pH-dependent and carrier-mediated transport has been shown, an intestinal pyridoxine transporter is yet to be identified in any mammalian species (11). After the absorption of free vitamin B6, it enters the blood and is converted back to PLP or PMP.

Microbial vitamin B6 is synthesized as PLP from deoxyxylulose 5-phosphate and 4-phosphohydroxy-L-threonine or from glyceraldehyde-3-phosphate and d-ribulose 5-phosphate (10). In the large intestine, bacteria-derived PLP is converted to free vitamin B6, which is absorbed by passive transport, transported to the blood, and distributed throughout the body.

Metagenomic analysis has shown that *Bacteroides fragilis* and *Prevotella copri* (Bacteroidetes), *Bifidobacterium longum* and, *Collinsella aerofaciens* (Actinobacteria), and *Helicobacter pylori* (Proteobacteria) possess a vitamin B6 biosynthesis pathway. Bacteroidetes and Proteobacteria likely produce vitamin B6 starting from deoxyxylulose 5-phosphate and 4-phosphohydroxy-l-threonine, whereas Actinobacteria likely start from glyceraldehyde-3-phosphate and d-ribulose 5-phosphate. In contrast, most Firmicutes genera (*Veillonella, Ruminococcus, Faecalibacterium*, and *Lactobacillus* spp.), except for some *Clostridium* (*C. bartlettii, C. leptum, C. methylpentosum*, and *C. sporogenes*) and *Lactobacillus* spp. (*L. brevis* and *L. ruminis*) lack a vitamin B6 biosynthesis pathway (10) (Table 1).

## Vitamin B7

Vitamin B7 (biotin) is a cofactor for several carboxylases that are essential for glucose, amino acid, and fatty acid metabolism (89). For example, vitamin B7 is an essential cofactor for acetyl-CoA carboxylase and fatty acid synthase, which are enzymes involved in fatty acid biosynthesis (90, 91). Thus, vitamin B7 likely influences immunometabolism. WHO/FAO recommends a daily vitamin B7 intake of 30 μg for adults (16). Vitamin B7 suppresses gene expression by binding to (biotinylating) histones; these genes include that encoding NF-κB, which is a major signaling molecule for the production of several pro-inflammatory cytokines (e.g., tumor necrosis factor alpha, IL-1, IL-6, IL-8) (92, 93). Nuclear transcription of NF-κB is activated in response to vitamin B7 deficiency (94), suggesting that biotinylation of histones suppresses the expression of genes encoding pro-inflammatory cytokines in NF-κB signaling (Figure 3). Therefore, vitamin B7 has anti-inflammatory effects by inhibiting NF-κB activation, and dietary vitamin B7 deficiency causes inflammatory responses via enhanced secretion of pro-inflammatory cytokines (95, 96).

Vitamin B7 is abundant in foods such as nuts, beans, and oilseed. However, raw egg-white contains a large amount of avidin, which binds strongly to vitamin B7 and prevents its absorption in the gut (97). Therefore, vitamin B7 deficiency can be caused not only by insufficient vitamin B7 intake, but also by excessive intake of raw egg-white. Dietary biotin exists as a free protein-bound form or as biocytin (11). In the small intestine, biotinidase releases free biotin from the bound protein and the free biotin is absorbed via the biotin transporter SMVT (98).

Vitamin B7 is also produced by intestinal bacteria as free biotin synthesized from malonyl CoA or pimelate via pimeloyl-CoA (99, 100). Bacterial free biotin is absorbed by SMVT expressed in the colon (23, 101). Metagenomic analysis has shown that *Bacteroides fragilis* and *Prevotella copri* (Bacteroidetes); *Fusobacterium varium* (Fusobacteria) and *Campylobacter coli* (Proteobacteria) possess a vitamin B7 biosynthesis pathway (10). In contrast, *Prevotella* spp. (Bacteroidetes), *Bifidobacterium* spp. (Actinobacteria), and *Clostridium, Ruminococcus, Faecalibacterium*, and *Lactobacillus* spp. (Firmicutes) lack such a pathway (Table 1); however, they do express free biotin transporter (10, 102), suggesting that these bacteria also utilize dietary and bacterial vitamin B7 and therefore may compete with the host. Thus, free biotin may influence the composition of the gut microbiota, because biotin is necessary for the growth and survival of the microbiota. Indeed, biotin deficiency leads to gut dysbiosis and the overgrowth of *Lactobacillus murinus*, leading to the development of alopecia (103). Furthermore, vitamin B7 production appears to proceed in a cooperative manner among different intestinal bacteria; *Bifidobacterium longum* in the intestine produces pimelate, which is a precursor of vitamin B7 that enhances vitamin B7 production by other intestinal bacteria (104).

## Vitamin B9

Vitamin B9 (folate), in its active form as tetrahydrofolate, is a cofactor in several metabolic reactions, including DNA and amino acid synthesis. WHO/FAO recommends a daily vitamin B9 intake of 400 μg for adults (16). Owing to the high requirement of vitamin B9 by red blood cells, vitamin B9 deficiency leads to megaloblastic anemia (23). Vitamin B9 deficiency also inhibits the proliferation of human CD8^+^ T cells *in vitro* by arresting the cell cycle in the S phase and increasing the frequency of DNA damage (105). Moreover, vitamin B9 contributes to the maintenance of immunologic homeostasis. Regulatory T cells (Treg) express high levels of vitamin B9 receptor (folate receptor 4 [FR4]). Administration of anti-FR4 antibody results in specific reduction in the Treg cell population (106), suggesting that the vitamin B9–FR4 axis is required for Treg cell maintenance. *In vitro* culture of Treg cells under vitamin B9-reduced conditions leads to impaired cell survival, with decreased expression of anti-apoptotic Bcl2 molecules, although naïve T cells retain the ability to differentiate into Treg cells; this suggests that vitamin B9 is a survival factor for Treg cells (87). Consistent with these findings, deficiency of dietary vitamin B9 results in reduction of the Treg cell population in the small intestine (107, 108). Since Treg cells play an important role in the prevention of excessive immune responses (109), mice fed a vitamin B9-deficient diet exhibit increased susceptibility to intestinal inflammation (107).

Foods such as beef liver, green leafy vegetables, and asparagus contain high levels of vitamin B9. Vitamin B9 exists as both mono- and polyglutamate folate species in the diet (110). Folate polyglutamate is deconjugated to the monoglutamate form and then absorbed in the small intestine via folate transporters such as proton-coupled folate transporter (PCFT) (11, 111). In the intestinal epithelium, folate monoglutamate is converted to tetrahydrofolate (THF), an active form and cofactor, before being transported to the blood (111).

Intestinal bacteria synthesize vitamin B9 as THF from GTP, erythrose 4-phosphate, and phosphoenolpyruvate (38, 112). Bacterial THF is directly absorbed in the colon via PCFT and distributed through the body by the blood (113). Metagenomic analysis has shown that *Bacteroides fragilis* and *Prevotella copri* (Bacteroidetes); *Clostridium difficile, Lactobacillus plantarum, L. reuteri, L. delbrueckii* ssp. *bulgaricus*, and *Streptococcus thermophilus* (Firmicutes), some species in *Bifidobacterium* spp (Actinobacteria); *Fusobacterium varium* (Fusobacteria) and *Salmonella enterica* (Proteobacteria) possess a folate biosynthesis pathway (Table 1) (10, 40). This suggests that almost all species in all phyla produce folate. Indeed, dietary supplementation with *Bifidobacterium* probiotic strains (*B. adolescentis* and *B. pseudocatenulatum*) enhances folate production in the large intestine of folate-deficient rats and folate-free culture medium (38, 41, 114). Furthermore, *Lactobacillus plantarum, L. delbrueckii* ssp. *bulgaricus*, and *L. reuteri* enhance folate production in bacterial culture supernatant lacking the components needed for folate synthesis (38, 39, 115).

In commensal bacteria, a vitamin B9 metabolite, 6-formylpterin (6-FP), is produced by photodegradation of folic acid (116). Like the vitamin B2 metabolite 6-hydroxymethyl-8-d-ribityllumazine, 6-FP binds to MR1, but unlike 6-hydroxymethyl-8-d-ribityllumazine it cannot activate MAIT cells (62, 117). An analog of 6-FP, acetyl-6-FP, is an antagonist of MR1, which inhibits MAIT cell activation (118). As mentioned in the section on vitamin B2, 6-hydroxymethyl-8-d-ribityllumazine activates MAIT cells, which provide defense against pathogens, so vitamin B9 metabolites may suppress excess MAIT cell responses and prevent excessive allergic and inflammatory responses (Figure 2). The quantitative balance between dietary vitamin B2 and B9 and the composition of the microbiota and its ability to metabolize these vitamins may be keys to understanding MAIT-cell-mediated homeostasis in the intestine.

## Vitamin B12

Vitamin B12 (cobalamin) is a cobalt-containing vitamin that, in its active forms of methylcobalamin and adenosylcobalamin, catalyzes methionine synthesis (119). WHO/FAO recommends a daily vitamin B12 intake of 2.4 μg for adults (16). Together with vitamin B6 and B9, vitamin B12 plays important roles in red blood cell formation and nucleic acid synthesis, especially in neurons. Therefore, vitamin B12 deficiency causes megaloblastic anemia and nervous system symptoms such as numbness of the hands and feet (119). In terms of host immunity, dietary vitamin B12 deficiency decreases the number of CD8^+^ T cells and suppresses natural killer T-cell activity in mice; supplementation with methylcobalamin improves these conditions (120), suggesting that vitamin B12 contributes to the immune response via CD8^+^ T cells and natural killer T cells.

Beef liver, bivalves, fish, chicken, and eggs contain high levels of vitamin B12. Dietary vitamin B12 exists in complex with dietary protein and is decomposed to free vitamin B12 by pepsin in the stomach. Free vitamin B12 is absorbed by the epithelial cells of the small intestine via intrinsic factor (IF), a gastric glycoprotein. Inside the epithelial cells, IF-vitamin B12 complex is decomposed to free vitamin B12 by lysosome and then released into the blood, where it is converted to the active form and distributed throughout the body (121, 122).

Bacterial vitamin B12 is synthesized from precorrin-2 to produce adenosylcobalamin (10), which is absorbed directly by the large intestine and distributed throughout the body; the mechanism underlying this absorption is currently unclear. Metagenomic analysis has predicted that *Bacteroides fragilis* and *Prevotella copri* (Bacteroidetes); *Clostridium difficile, Faecalibacterium prausnitzii* and *Ruminococcus lactaris* (Firmicutes); *Bifidobacterium animalis, B.infantis*, and *B.longum* (Actinobacteria); *Fusobacterium varium* (Fusobacteria) possess a vitamin B12 biosynthesis pathway (Table 1) (10, 32, 42, 43, 45). Indeed, *Lactobacillus plantarum* and *L. coryniformis* isolated from fermented food produce vitamin B12 (33), and *Bifidobacterium animalis* synthesizes vitamin B12 during milk fermentation (123).

## Conclusion

**Figure 4:**
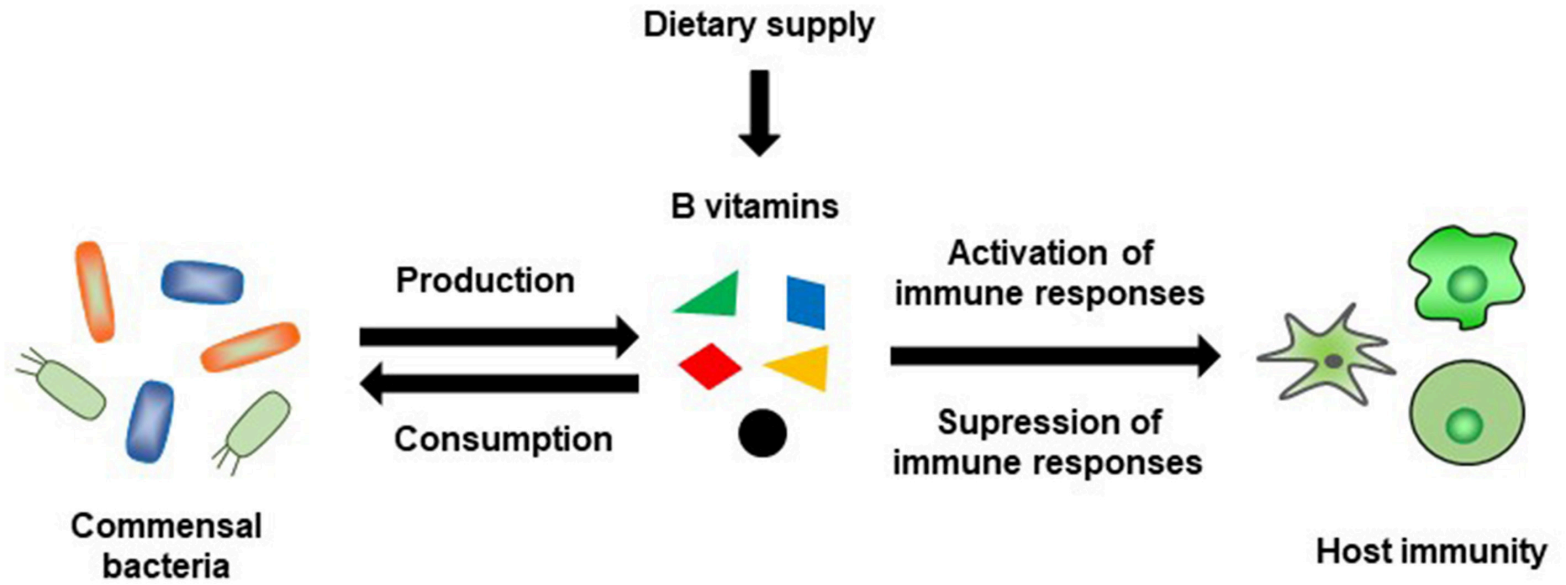
Schematic representation of B-vitamin-mediated interaction between commensal bacteria and host immunity.

B-vitamin-mediated immunological regulation is specific to different immune cells and immune responses: that is, different B vitamins are required for different immune responses (Figure 4). It was once thought that B vitamins were obtained only from the diet; however, we know now that this is not the case and that the intestinal microbiota is also an important source of vitamins. Within the intestinal microbiota, not all bacteria produce B vitamins and some bacteria utilize dietary B vitamins or B vitamins produced by other intestinal bacteria for their own needs; therefore, there may be competition between the host and the intestinal microbiota for B vitamins (Figure 4). Research in this field is complicated, because not only does the composition of the intestinal microbiota vary among individuals, but also the composition of the diet can alter both the composition and function of the intestinal microbiota. Therefore, vitamin-mediated immunological maintenance also varies among individuals. Further examinations in this field are needed, and the new information uncovered will help to develop a new era of precision health and nutrition.

## Author Contributions

KY and KH wrote the draft of review article which was corrected by JK. KY, KH, and KS drew figures and JK performed correction.

### Conflict of Interest Statement

KS was employed by Nippon Flour Mills Co., Ltd. The remaining authors declare that the research was conducted in the absence of any commercial or financial relationships that could be construed as a potential conflict of interest.
